# Frequent callers contacting the Norwegian national emergency medical number 113: a retrospective study

**DOI:** 10.1186/s13049-024-01275-1

**Published:** 2024-10-14

**Authors:** Sara Naess Viken, Lars Myrmel, Guttorm Brattebø

**Affiliations:** 1 Department of Clinical Medicine, University of Bergen, Bergen, Norway; 2 Department of Anaesthesia & Intensive Care, Haukeland University Hospital, Bergen, Norway; 3 Norwegian National Advisory Unit on Emergency Medical Communication (KoKom), Haukeland University Hospital, Bergen, Norway

**Keywords:** Ambulances, Retrospective studies, Communication, Norway, Delivery of health care, Hospitals

## Abstract

**Background:**

Calling for help is the first link in the chain of survival; however, few studies have investigated the challenges faced by frequent callers (FCs) to emergency medical communication centres (EMCCs). This study aimed to explore the characteristics of FCs and the nature of their calls to the Bergen EMCC, Norway.

**Methods:**

This was a retrospective analysis of all emergency calls to the Bergen EMCC over three consecutive years (2019–2021). Bergen is the second-largest city in Norway, and the Bergen EMCC and ambulance services are part of the specialist medical service, covering a population of 460,000. The Bergen EMCC receives approximately 60,000 emergency calls per year. The study population comprised all adults identified during emergency medical calls. FCs were defined as individuals who registered five or more calls over 12 consecutive months during the three-year period.

**Results:**

The analysis included approximately 50,000 individuals, who made > 90,000 calls during the study period. Of those, 1,594 (3.2%) were FCs, accounting for approximately one in four (21,339 of 90,085, 23.7%) calls. The FCs included more men (882 of 1,594 (55.3%) vs. 24,204 of 47,564 (50.9%)) and registered a lower proportion of calls with an acute degree of urgency (6,051 of 21,339 calls (28.4%) vs. 30,276 of 68,746 calls (44.0%)). Calls from FCs showed an even occurrence throughout the week, peaking between 19:00 h and 20:00 h. Compared with calls from non-FCs, calls from FCs had a higher proportion of ‘no response/verbal referral to local emergency medical department’ and involved a lower proportion of hospital transfers. The EMCC most frequently used the medical criterion ‘Mental health problems/suicide’ for calls from FCs.

**Conclusions:**

FCs were common, and more often men. The EMCC dispatched ambulances or admitted patients to hospitals less frequently following these calls. Many of these situations could be handled in other parts of the healthcare system, reducing the burden on EMCCs, and providing more suitable services for FCs. Thus, EMCCs should identify and adjust patient management to match their actual needs.

**Supplementary Information:**

The online version contains supplementary material available at 10.1186/s13049-024-01275-1.

## Background

A small group of callers make numerous calls to the medical communication centres (EMCCs), sometimes for trivial reasons. These may be referred to as ‘frequent callers’ (FCs). Some of these callers call the emergency number after not receiving suitable services from other parts of the healthcare system, e.g. mental health services. Managing such calls from FCs may reduce the availability of responders to other callers requiring immediate intervention, thereby impairing an EMCC’s ability to provide acute help to anyone in need (including other FCs).

Several studies have examined frequent users of emergency departments (EDs) and identified certain characteristics of this group. This group has been shown to be heterogeneous in nature and more likely to include patients with chronic diseases, mental illnesses, or substance use disorders [1–9]. However, the definition of FCs varies across studies [2, 5, 7, 10]. One definition identified frequent users as those with more than four visits to the ED per year [2]. However, few studies have focused on FCs, and the generalizability of the findings for frequent ED users to FCs remains to be determined. Because FCs may have a significant impact on the functioning of EMCCs, both in terms of time consumption and costs, more knowledge of the characteristics of FCs is essential [11]. By examining the characteristics of this relatively small but challenging group, we aimed to explore whether these callers showed features distinguishing them from non-frequent callers (non-FCs). Therefore, this study aimed to explore the characteristics of FCs and the nature of their calls to the Bergen EMCC over a period of three consecutive years.

## Methods

### Study design and setting

In Norway, 16 EMCCs serve the national emergency number 113. Calls to this number are automatically routed to the nearest EMCC. The operators are specially trained nurses and ambulance personnel who determine the type of help required and the degree of urgency in each case [12]. The majority of individuals calling 113 only make one or few such calls per year. This study was a retrospective analysis of routinely collected data from the electronic record system (acute medical information system, AMIS) used in all EMCCs in Norway [13]. We used data obtained from the Bergen Health Trust, which serves the second-largest city in the country, over three consecutive years. The Bergen EMCC serves a population of over 460,000 inhabitants and receives approximately 60,000 emergency calls each year [14]. For each call and/or patient, specific data are registered in the AMIS; these include the call origin; date; time; place of incident; degree of urgency; and caller’s/patient’s name, age, and address. Caller data are not collected when the caller is not a patient. In addition, the EMCC operator registers the chief complaint or problem for each call and assigns a criterion based on the Norwegian Index for Medical Emergency Assistance (Index) [15, 16]. The Index is a national decision support system used by all Norwegian EMCC operators to prioritise and manage calls; it contains a start page and 40 symptom-based ‘cards’ along with eight administrative ‘cards’. The Index criteria are not meant to be used to diagnose the patient via a phone call, but rather to determine the type of response and urgency level required in each case.

### Data collection

**Fig. 1:**
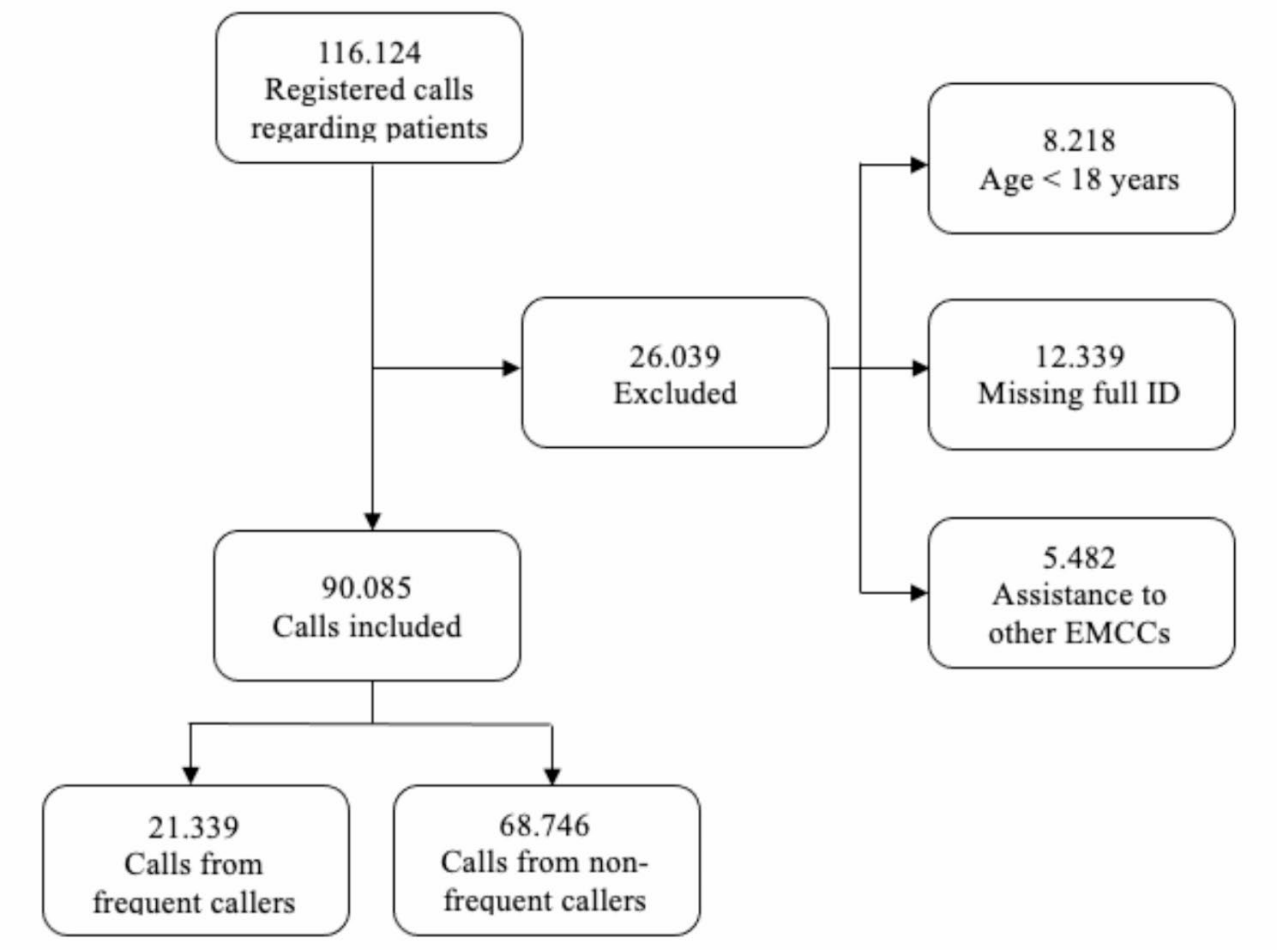
Flowchart of the inclusion process

This study included all emergency calls to the Bergen EMCC through 113, with available patient demographics, from January 1 2019 to December 31 2021. Calls that could not be linked to an individual patient (i.e. calls that were not registered with a unique social security number (ID)) were excluded. Administrative calls (e.g. calls from fire and rescue services, police, and other EMCCs) and calls from people under 18 years of age were also excluded. (There are stricter regulations on data concerning children under the age of 18.) Thus, a total of 90,085 calls made over the three-year period were included in this study (Fig. 1).

### Data management and statistical analysis

The total number of registered calls for each patient was calculated based on all incidents registered with an ID during the study period. Based on the ID, the patients’ sex and age were registered before a de-identifiable data file was used for analysis and stored on the hospital’s secured data server.

FCs were defined as those who made five or more registered calls to the EMCC from a unique ID over 12 consecutive months during the period from 2019 to 2021, consistent with other studies on FCs [17–19]. Accordingly, non-FCs represented the rest of the data population. Owing to data privacy regulations, callers could only be characterised by age and sex. The age was calculated from the time of the first call.

All records (several for some of the patients) were analysed for the type of calls, time of event (weekday and hour of the day), chief complaint for calling according to the Index criteria, degree of urgency, and responses [i.e. no response/verbally referred to the local emergency medical department (LEMD), transport to hospital, transport to the LEMD/general practitioner (GP), treat and release (non-conveyance), transport to other institutions, or transport to nursing homes]. The degree of urgency for each call was registered as ‘acute’, ‘urgent’, or ‘non-urgent’ in the AMIS. These were divided into two groups for the analysis: ‘acute’ and ‘non-acute’ (with ‘non-acute’ including ‘urgent’ and ‘non-urgent’). The call date and time were obtained from the AMIS, and weekdays were calculated using this information.

To visualise patterns at the time of contact, the distribution of calls throughout the week and time of day were graphed. Time of day represents the number of calls in the preceding hour (the number of calls presented at 08:00 h indicates calls made between 07:00 h and 08:00 h). The proportion of calls from FCs was calculated for each day of the week and every hour of the day. The five most common Index criteria for the two groups were compared. Any obvious errors in criterion use were corrected. For each year of the study period, the total number of incidents and the proportion of FCs were compared to reveal variations in activity over the three years, since the data collection period included periods both before and during the coronavirus disease 2019 pandemic.

Data were analysed using IBM SPSS, version 26 (IBM Corp., Armonk, New York, USA) to characterise the FCs, call time, number of calls, and measures undertaken for each incident. The median, range, and interquartile range (IQR; 25th and 75th percentiles) were calculated for age. Continuous variables were evaluated using the *t* test, and categorical data were compared using the chi-squared test. A *p*-value of < 0.05 was considered to indicate significance.

## Results

Overall, 49,158 callers made 90,085 calls over the three-year study period. Of these, 1,594 (3.2%) callers who made 21,339 (23.7%) calls were defined as FCs.

### Calls per individual ID

**Fig. 2:**
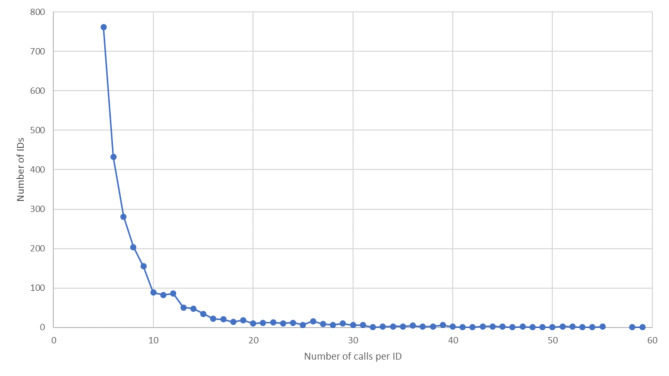
Number of calls per individual unique social security number (2,475 individuals who made 5–60 calls each) Not included in the graph are 35 individuals who made 61–458 calls each and 46,648 individuals who made 1–4 calls each

Figure 2 shows the number of individual IDs registered with 5–60 calls each during the study period. Individuals with 1–4 registered calls were omitted from the graph for better visualisation, and a minimum of five registered calls during the period were required for the caller to be defined as an FC.

### Degree of urgency

In assessments of the degree of urgency registered for each call, the proportion of acute calls from FCs was 28.4% (6,051 of 21,339); this was significantly lower (*p* < 0.05) than that from non-FCs (44.0%; 30,276 of 68,746). The total proportion of acute calls was 40.3% (36,327 of 90,085).

### Sex and age

More than half of the FCs were men (882 of 1,594 (55.3%)); this proportion was significantly higher (*p* < 0.05) than the proportion of men among non-FCs (24,204 of 47,564 (50.9%)). The mean age of the FCs was 57.6 years (range, 18–99 years; IQR, 38–76 years), which was significantly higher (*p* < 0.05) than that of the non-FCs (mean age, 55.5 years; range, 18–105 years; IQR, 34–75 years). The median ages of the FCs and non-FCs were 61 years and 57 years, respectively.

### Distribution of calls throughout the week

**Fig. 3:**
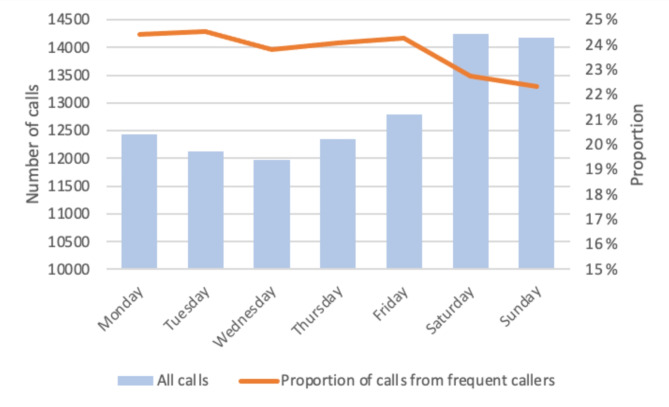
Call distribution throughout the week and proportion of calls from frequent callers on each weekday

The number of calls from FCs showed little variation throughout the week, with the lowest number observed on Wednesdays (*n* = 2,848) and the highest on Saturdays (*n* = 3,240). The median number of calls per day was 3,035. Furthermore, the proportion of calls from FCs was approximately 24% throughout the week, with a slight decrease on Saturdays and Sundays, as shown in Fig. 3. However, the total number of calls increased on weekends.

### Distribution of calls throughout the hours of the day

**Fig. 4:**
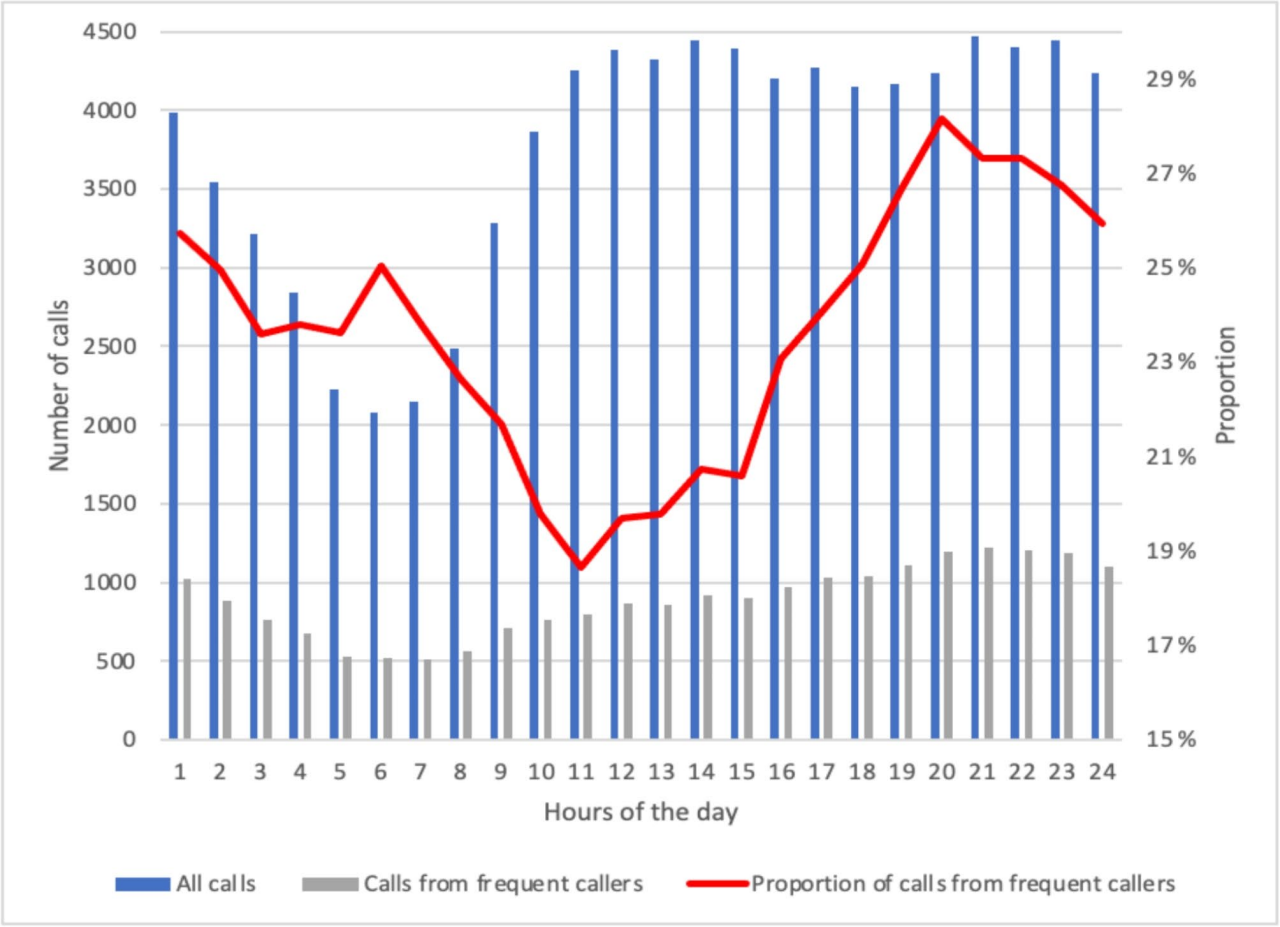
Distribution of calls throughout the hours of the day Figure shows the total number of calls, number of calls from frequent callers, and proportion of calls from frequent callers within each hour

Figure 4 shows the calls received during the hours of the day, including those from FCs and the total population. Throughout the day, the total number of calls increased gradually from the lowest at 05:00–06:00 h to 10:00–11:00 h. From 11:00 h to 24:00 h, the total number of calls remained more or less constant. The proportion of calls from FCs showed a small peak at 05:00–06:00 h (25%) and was the lowest at 10:00–11:00 h (19%); thereafter, it increased gradually, increasing during 15:00–20:00 h and peaking at 19:00–20:00 h (28%).

### Responses to the calls

**Fig. 5:**
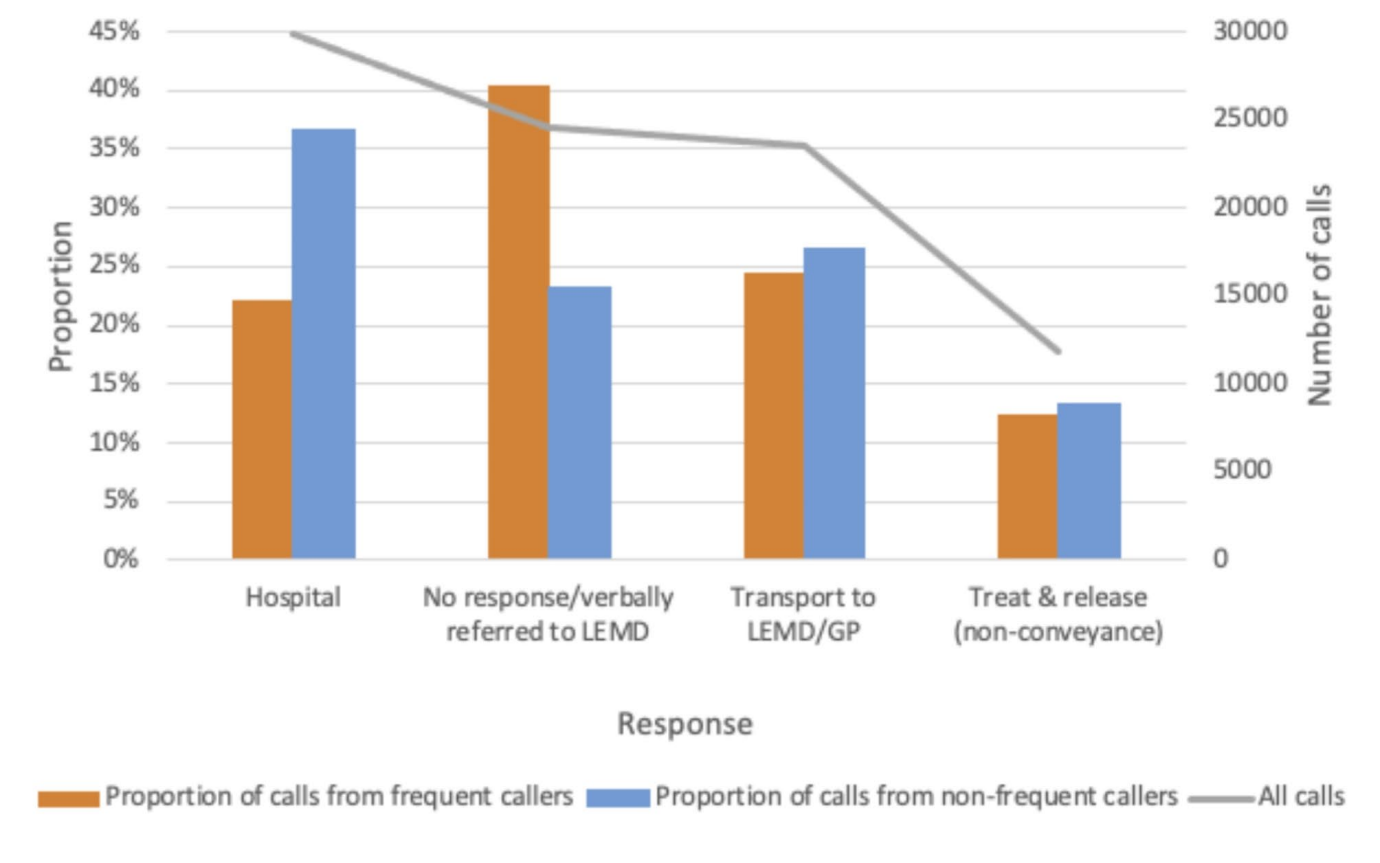
Responses to the calls All calls and the proportions of calls from frequent callers and non-frequent callers that involved transport to a hospital, no response/verbal referral to local emergency medical department, transport to local emergency medical department/general practitioner, and treat & release (non-conveyance)

Figure 5 shows that calls from FCs less frequently resulted in transport to hospital (22.1%; *n* = 4,722) as compared with calls from non-FCs (36.7%; *n* = 25,196). Additionally, the action ‘No response/verbally referred to LEMD’ was registered in 40.4% (*n* = 8,612) of the calls from FCs and 23.3% (*n* = 15,984) of the calls from non-FCs; this difference was significant (*p* < 0.05). The measure ‘Others’, which included transport to other institutions, disrupted calls, and transport to nursing homes, accounted for a small number of calls (*n* = 296) and was not included in the figure.

### Most frequently used index criteria

As shown in Table 1, ‘Mental health problems/suicide’ was the Index criterion most frequently used for calls from FCs. However, this criterion was the eighth-most frequent criterion for calls from non-FCs [see Additional file 1]. Among FCs, the five most frequently used Index criteria accounted for 70.1% (*n* = 14,965) of all calls. In contrast, among non-FCs, the top five Index criteria accounted for 53.9% (*n* = 37,043) of the total number of calls.

**Table 1:** The five most frequent index criteria registered as the chief complaint at the time of contact with the EMCC (number of calls,* proportion of total number of calls for each group)*

The five most frequently used Index criteria	Calls from frequent callers *n* = 21,339	Calls from non-frequent callers *n* = 68,746	All calls *n* = 90,085
1	Mental health problems/suicide (*n* = 4,418, 20.7%)	Unidentified problem* (*n* = 9,440, 13.7%)	Unidentified problem* (*n* = 13,579, 15.1%)
2	Unidentified problem* (*n* = 4,139, 19.4%)	Chest pain/cardiac disease (*n* = 9,124, 13.3%)	Chest pain/cardiac disease (*n* = 11,226, 12.5%)
3	Intoxication/overdose (*n* = 2,520, 11.8%)	Fractures/wounds/minor injuries (*n* = 6,721, 9.8%)	Mental health problems/suicide (*n* = 8,131, 9.0%)
4	Chest pain/ cardiac disease (*n* = 2,102, 9.9%)	Abdominal or back pain (*n* = 6,274, 9.1%)	Fractures/wounds/minor injuries (*n* = 7,524, 8.4%)
5	Breathing problems/shortness of breath (*n* = 1,786, 8.4%)	Transport arrangements (*n* = 5,484, 8.0%)	Abdominal or back pain (*n* = 7,376, 8.2%)
	Other criteria (*n* = 6,374, 29.9%)**	Other criteria (*n* = 31,703, 46.1%)**	Other criteria (*n* = 42,249, 46.9%)**

* Unidentified problem: This criterion was used when the caller could not explain the chief complaint or the call taker could not find other suitable criteria.

** See supplemental file

## Discussion

We found that 1,594 (3.2%) individuals were FCs, who accounted for 21,339 (23.7%) of the calls to the emergency medical number. One study revealed that frequent ED users constituted 4.5–8% of all ED patients and accounted for 21–28% of all visits [5]. Other studies have found that frequent ED users constituted 3.1–3.7% of all ED users and accounted for 12.2–13.8% of all ED visits [2, 20, 21]. Our FCs accounted for a larger proportion of calls to the EMCC than those seen in studies on frequent ED users, suggesting that FCs have a lower threshold for contacting the healthcare system than frequent ED users. However, this also implies that EMCCs most likely can avoid unnecessary ED admissions.

Previous studies on the frequent use of healthcare services have tended to focus on frequent ED users, and research exploring the characteristics of both frequent users of emergency medical services (EMSs) and FCs to the EMSs is limited [10]. Consequently, limited data were available to compare with the findings of the present study. The applicability of the findings for frequent ED users to FCs remains to be examined.

Compared with non-FCs, FCs showed a lower proportion of calls being categorised as ‘acute’. This could mean that a larger proportion of calls from FCs are concerned with less urgent issues, which may limit the EMCC’s ability to provide help to those in need of acute emergency services. Some of these non-acute incidents can be handled by other parts of the healthcare system, such as GPs or other healthcare services. Although previous studies on frequent ED users have indicated that they tend to use other healthcare services more frequently than non-frequent users [1, 4, 5, 9, 22, 23], the applicability of these findings to the FCs in our study remains to be ascertained. Interestingly, compared with the non-FCs, the FCs in our study included a higher proportion of men and exhibited a significantly higher mean age. The finding of a larger proportion of men and elderly among FCs is perhaps not that surprising, since men and elderly are more comorbid, and perhaps five contacts to the EMCC may be expected for some cases.

The proportion of calls from FCs showed little variation throughout the week but showed a slight drop on Saturdays and Sundays, since the proportion of calls from non-FCs was greater than that from FCs on these days. The proportion of calls from FCs seemed to remain steady throughout the week. During the day, the largest proportion of calls from FCs was outside daytime hours, coinciding with the reduced availability of other healthcare and social services that FCs may need. This suggests that when other services, such as primary and community care services, are available, the number of calls from FCs decline [24]. This phenomenon has also been observed among frequent ED users [25].

‘No response/verbally referred LEMD’ was the measure assigned to the largest proportion of calls from FCs; fewer calls from non-FCs were assigned this measure. In comparison with calls from FCs, a significantly higher proportion of calls from non-FCs resulted in transport to a hospital.

Frequent users of EDs arrive by ambulance more often and are more likely to be admitted to hospitals [2, 6, 10, 26, 27]. In our population, nearly one in two calls from FCs resulted in ambulance transport, either to the hospital or the LEMD/GP. One limitation of previous studies on callers to EMSs is that the majority of these studies focused on patients being transported by ambulance, excluding those whose calls are not conveyed or sent a response [24]. Most calls from FCs in our study did not involve the ambulance service, highlighting the importance of including all calls to examine the characteristics of this group. Since only half of the calls from FCs ended in transport to hospitals/LEMDs, the applicability of the characteristics of frequent ED users to FCs to EMCCs is uncertain.

The five most frequent Index criteria differed between the FCs and non-FCs. ‘Mental health problems/suicide’ was the most frequent criterion used for calls from FCs, whereas this criterion was outside of the top five criteria for calls from non-FCs. This corresponds to the findings of other studies on frequent ED users, where frequent ED users were more likely to have mental health diagnoses or present with psychiatric problems [2, 7, 28]. One study also found that FCs often experienced loneliness, social isolation, and a low quality of life [17]. Since one in four calls are from FCs, our findings indicate a need to establish other services that could better meet the needs of FCs. This could ease the pressure on EMCCs and optimise their functioning, and more importantly, improve medical services for FCs. This could be achieved by actively identifying and monitoring these patients in the EMCC activity data, to find solutions to their individual needs. The EMCCs could for example start with identifying the patients making > 30 calls per year – in our material this corresponds to 95 patients. To be able to establish a routinely attention and test various measures to reduce the number of FCs, more in-depth studies are needed to explore this group of patients. However, this was outside the scope of our study.

### Strengths and limitations

Since the study was based on routinely collected EMCC data from a population of 460,000 over a period of three consecutive years, data loss was limited. The use of consecutive 12-month periods over a 3-year period to define FCs helped identify more FCs than that possible with definitions restricted to calendar years.

Our study had some limitations as well. First, this study was based on a healthcare system that may differ from the systems in other countries. The Norwegian emergency healthcare system is a unique two-tiered system consisting of hospitals, EDs, and ambulance services along with a well-organised system of LEMDs in each municipality. Most contact with the emergency healthcare system was via phone calls, either to the EMCC or directly to the LEMD. The operators in the different centres cooperate and transmit calls in both ways, if needed. Since this structure differs from systems in other countries, the comparability of frequent users/callers to different systems may be challenging, and these findings may not be generalisable to other national systems. However, we believe that appropriate management of FCs is a common challenge regardless of the system.

Second, no standard definition of a frequent user of the ED or an FC to the EMS has been proposed to date. We used a definition of five or more incidents according to other studies on FCs [17–19]. In addition, research on FCs to emergency healthcare systems is also limited, and the applicability of the definition of frequent ED users to FCs remains to be ascertained.

Third, data regulations precluded the collection of more information about individual patients, and we could not evaluate the outcomes after the calls were managed by the EMCC operators. Thus, we do not know if the patients being transported or verbally referred to the LEMD were admitted to a hospital after visiting the LEMD or if the patients being transported to the hospital returned home without any specific diagnostics or treatment.

Finally, the EMCC operators registered only the chief complaints in each call. We do not know if several issues were presented and considered or whether the registered chief complaint was the real reason for calling. Therefore, the explanation for why some individuals are FCs may be more complex than could be inferred from a registered single chief complaint.

In the present study, we defined FCs as those who had made five or more calls within 12 consecutive months during the three-year study period. Consequently, a caller could call up to 12 times without being defined as an FC if the 12 calls were distributed as less than five calls within separate 12-months consecutive periods. Compared with definitions restricted to one calendar year, our definition likely identified more FCs to the EMCC.

## Conclusions

FCs accounted for a significant proportion of EMCC calls and were more frequently men. These calls were less frequently acute and often resulted in no EMS transport. While FCs occasionally called 113 because of the acute need for healthcare services, a large proportion of these calls should rather be handled in other parts of the healthcare system. This could free EMCC resources to help callers in need of urgent help, and also improve the quality of medical services for FCs. Thus, EMCCs would probably benefit from regularly analysing their own data and identifying these patients. Additional research is needed to better characterise and predict FCs and further develop the specific management of these patients to meet their actual medical needs.

## Electronic supplementary material

Below is the link to the electronic supplementary material.

**Supplementary Material 1:** Extended table of chief complaints from the Norwegian Index for Medical Emergency Assistance (Index)

## Data Availability

No datasets were generated or analysed during the current study.

## Abbreviations

AMIS: Acute medical information system
ED: Emergency department
EMCC: Emergency medical communication centre
EMS: Emergency medical service
FC: Frequent caller
GP: General practitioner
ID: Social security number
Index: Norwegian Index for Medical Emergency Assistance
IQR: Interquartile range
LEMD: Local emergency medical department
non-FC: Non-frequent caller

## References

[1] Moe J, O’Sullivan F, McGregor MJ, Schull MJ, Dong K, Holroyd BR, et al. Characteristics of frequent emergency department users in British Columbia, Canada: a retrospective analysis. CMAJ Open. 2021;9:E134–41. 33653768 10.9778/cmajo.20200168 PMC8034376

[2] Locker TE, Baston S, Mason SM, Nicholl J. Defining frequent use of an urban emergency department. Emerg Med J. 2007;24:398–401. 17513534 10.1136/emj.2006.043844 PMC2658272

[3] Pines JM, Asplin BR, Kaji AH, Lowe RA, Magid DJ, Raven M, et al. Frequent users of emergency department services: gaps in knowledge and a proposed research agenda. Acad Emerg Med. 2011;18:e64–9. 21676051 10.1111/j.1553-2712.2011.01086.x

[4] Hunt KA, Weber EJ, Showstack JA, Colby DC, Callaham ML. Characteristics of frequent users of emergency departments. Ann Emerg Med. 2006;48:1–8. 16781914 10.1016/j.annemergmed.2005.12.030

[5] LaCalle E, Rabin E. Frequent users of emergency departments: the myths, the data, and the policy implications. Ann Emerg Med. 2010;56:42–8. 20346540 10.1016/j.annemergmed.2010.01.032

[6] Fuda KK, Immekus R. Frequent users of Massachusetts emergency departments: a statewide analysis. Ann Emerg Med. 2006;48:9–16. 16781915 10.1016/j.annemergmed.2006.03.001

[7] Soril LJ, Leggett LE, Lorenzetti DL, Noseworthy TW, Clement FM. Characteristics of frequent users of the emergency department in the general adult population: a systematic review of international healthcare systems. Health Policy. 2016;120:452–61. 26947060 10.1016/j.healthpol.2016.02.006

[8] Krieg C, Hudon C, Chouinard MC, Dufour I. Individual predictors of frequent emergency department use: a scoping review. BMC Health Serv Res. 2016;16:594. 27765045 10.1186/s12913-016-1852-1 PMC5072329

[9] Doupe MB, Palatnick W, Day S, Chateau D, Soodeen RA, Burchill C, et al. Frequent users of emergency departments: developing standard definitions and defining prominent risk factors. Ann Emerg Med. 2012;60:24–32. 22305330 10.1016/j.annemergmed.2011.11.036

[10] Scott J, Strickland AP, Warner K, Dawson P. Frequent callers to and users of emergency medical systems: a systematic review. Emerg Med J. 2014;31:684–91. 23825060 10.1136/emermed-2013-202545

[11] Edwards MJ, Bassett G, Sinden L, Fothergill RT. Frequent callers to the ambulance service: patient profiling and impact of case management on patient utilisation of the ambulance service. Emerg Med J. 2015;32:392–6. 25312857 10.1136/emermed-2013-203496

[12] Øen TO, Juvkam PC, Aksnes AO, Jensen ÅC. Systemer Som Skal sikre god akuttmedisinsk praksis. In: Dreyer K, editor. Håndbok: Kommunikasjon Og samhandling i akuttmedisinske situasjoner. Bergen: KoKom; 2018. pp. 88–92.

[13] Helse Stavanger. Akuttmedisinsk informasjonssystem – AMIS. https://helsestavanger.arkivplan.no/layout/set/print/content/view/full/25062 (2006). Accessed 14 May 2024.

[14] Statistics Norway. https://www.ssb.no/ (2023). Accessed 14 May 2024.

[15] Ellensen EN, Hunskaar S, Wisborg T, Zakariassen E. Variations in contact patterns and dispatch guideline adherence between Norwegian emergency medical communication centres–a cross-sectional study. Scand J Trauma Resusc Emerg Med. 2014;22:2. 24398290 10.1186/1757-7241-22-2 PMC3892008

[16] NAKOS. Norsk indeks for Medisinsk Nødhjelp (NIMN) 4 utgave. 2018. https://www.nakos.no/pluginfile.php/1269/block_html/content/2019 engelske hjelpetekster NIMN 4 nav.pdf. Accessed 01 July 2024.

[17] Agarwal G, Lee J, McLeod B, Mahmuda S, Howard M, Cockrell K, et al. Social factors in frequent callers: a description of isolation, poverty and quality of life in those calling emergency medical services frequently. BMC Public Health. 2019;19:684. 31159766 10.1186/s12889-019-6964-1 PMC6547509

[18] Mahmuda S, Wade-Vallance A, Stosic A, Guenter D, Howard M, Agarwal G, et al. Understanding why frequent users of EMS call 9-1-1: a grounded theory study. Health Promot Pract. 2020;21:440–7. 30222003 10.1177/1524839918799504

[19] Hall MK, Raven MC, Hall J, Yeh C, Allen E, Rodriguez RM, et al. EMS-STARS: emergency medical services superuser transport associations: an adult retrospective study. Prehosp Emerg Care. 2015;19:61–7. 25093273 10.3109/10903127.2014.936630

[20] Giannouchos T, Pirrallo R, Ukert B. Factors associated with persistent multiyear frequent emergency department use. Emerg Med J. 2023;40:589–95. 37164623 10.1136/emermed-2022-212740

[21] Moe J, Bailey AL, Oland R, Levesque L, Murray H. Defining, quantifying, and characterizing adult frequent users of a suburban Canadian emergency department. CJEM. 2013;15:214–26. 23777993 10.2310/8000.2013.130936

[22] Hansagi H, Olsson M, Sjöberg S, Tomson Y, Göransson S. Frequent use of the hospital emergency department is indicative of high use of other health care services. Ann Emerg Med. 2001;37:561–7. 11385324 10.1067/mem.2001.111762

[23] Huang JA, Weng RH, Lai CS, Hu JS. Exploring medical utilization patterns of emergency department users. J Formos Med Assoc. 2008;107:119–28. 18285244 10.1016/S0929-6646(08)60125-4

[24] Scott J, Strickland AP, Warner K, Dawson P. Describing and predicting frequent callers to an ambulance service: analysis of 1 year call data. Emerg Med J. 2014;31:408–14. 23413152 10.1136/emermed-2012-202146

[25] Milbrett P, Halm M. Characteristics and predictors of frequent utilization of emergency services. J Emerg Nurs. 2009;35:191–8. 19446122 10.1016/j.jen.2008.04.032

[26] Markham D, Graudins A. Characteristics of frequent emergency department presenters to an Australian emergency medicine network. BMC Emerg Med. 2011;11:21. 22171720 10.1186/1471-227X-11-21 PMC3267650

[27] Jelinek GA, Jiwa M, Gibson NP, Lynch AM. Frequent attenders at emergency departments: a linked-data population study of adult patients. Med J Aust. 2008;189:552–6. 19012551 10.5694/j.1326-5377.2008.tb02177.x

[28] Giannouchos TV, Kum HC, Foster MJ, Ohsfeldt RL. Characteristics and predictors of adult frequent emergency department users in the United States: a systematic literature review. J Eval Clin Pract. 2019;25:420–33. 31044484 10.1111/jep.13137

