# Supplementary material for: Frequent callers contacting the Norwegian national emergency medical number 113: a retrospective study
Source: Scand J Trauma Resusc Emerg Med. 2024 Oct 14;32:102. doi: 10.1186/s13049-024-01275-1 (PMC11475646; doi:10.1186/s13049-024-01275-1)
Supplement: Supplementary file 1 — Supplementary Material 1: Extended table of chief complaints from the Norwegian Index for Medical Emergency Assistance (Index) [file 13049_2024_1275_MOESM1_ESM.docx]

**Supplemental material**

**Table 2.** *The Index criteria registered as chief complaint at the time of contact, listed after frequency (number of calls) for each caller group*

| **Frequency of Index criteria** | **Calls from frequent callers**  **n = 21,339** | **Calls from non-frequent callers**  **n = 68,476** | **All calls n = 90,085** |
| --- | --- | --- | --- |
| **6** | Abdominal or back pain (1,102) | Possible stroke/altered level of consciousness (4,777) | Transport arrangements (6,507) |
| **7** | Transport arrangements (1,023) | Breathing problems/shortness of breath (3,871) | Intoxication/overdose (5,977) |
| **8** | Fractures/wounds/minor injuries (803) | Mental health problems/suicide (3,713) | Breathing problems/shortness of breath (5,657) |
| **9** | Seizure/convulsions/fits (588) | Intoxication/overdose (3,457) | Possible stroke/altered level of consciousness (5,243) |
| **10** | Possible stroke/altered level of consciousness (466) | Fever/infection/sepsis (2,032) | Fever/infection/sepsis (2,355) |
| **11** | Fever/infection/sepsis (323) | Injury - possibly extensive/severe (1,948) | Injury - possibly extensive/severe (2,155) |
| **12** | Urinary tract (257) | Road traffic injury (1,347) | Seizure/convulsions/fits (1,667) |
| **13** | Diabetes (249) | Ear/nose/throat (1,189) | Ear/nose/throat (1,379) |
| **14** | Injury - possibly extensive/severe (207) | Seizure/convulsions/fits (1,079) | Road traffic injury (1,372) |
| **15** | Ear/nose/throat (190) | Headache (955) | Headache (1,140) |
| **16** | Headache (185) | Urinary tract (779) | Urinary tract (1,036) |
| **17** | Bleeding - non-traumatic (145) | Unresponsive adult - not breathing normally (764) | Bleeding - non-traumatic (893) |
| **18** | Unresponsive adult/child - breathing normally (142) | Bleeding - non-traumatic (748) | Diabetes (892) |
| **19** | Violence/abuse (123) | Diabetes (643) | Unresponsive adult - not breathing normally (846) |
| **20** | Skin/rash (112) | Unresponsive adult/child - breathing normally (624) | Unresponsive adult/child - breathing normally (766) |
| **21** | No criteria/response (87) | Allergic reaction (605) | Allergic reaction (653) |
| **22** | Unresponsive adult - not breathing normally (82) | Gynaecology/pregnancy (486) | Violence/abuse (596) |
| **23** | Allergic reaction (48) | Violence/abuse (473) | Gynaecology/pregnancy (531) |
| **24** | Gynaecology/pregnancy (45) | Skin/rash (380) | Skin/rash (492) |
| **25** | Poisoning - not alcohol/drug related (40) | Possible death/cot death (260) | Eye (279) |
| **26** | Eye (33) | Labour/child birth (258) | Possible death/cot death (274) |
| **27** | Burns/scalding/electrical injury (30) | Eye (246) | Labour/child birth (265) |
| **28** | Road traffic injury (25) | Burns/scalding/electrical injury (221) | Burns/scalding/electrical injury (251) |
| **29** | Choking/airway obstruction (foreign object in airway) (16) | Covid-19 (187) | No criteria/response (231) |
| **30** | Possible death/cot death (14) | Choking/airway obstruction (foreign object in airway) (160) | Covid-19 (198) |
| **31** | Covid-19 (11) | No criteria/response (144) | Choking/airway obstruction (foreign object in airway) (176) |
| **32** | Hypothermia (9) | Poisoning - not alcohol/drug related (131) | Poisoning - not alcohol/drug related (171) |
| **33** | Labour/child birth (7) | Animal bites/insect stings (63) | Animal bites/insect stings (67) |
| **34** | Animal bites/insect stings (4)  Hyperthermia/heat stroke (4) | Hypothermia (54) | Hypothermia (63) |
| **35** | Drowning (2) | Drowning (41) | Drowning (43) |
| **36** | Major disaster/mass-casualty incident (1)  Chemicals/gasses/  CBRNe (1) | Major disaster/mass-casualty incident (24) | Major disaster/mass-casualty incident (25) |
| **37** |  | Diving accident (19) | Diving accident (19) |
| **38** |  | Chemicals/gases/  CBRNe (14) | Chemicals/gases/  CBRNe (15)  Hyperthermia/heat stroke (15) |
| **39** |  | Hyperthermia/heat stroke (11) |  |
